# Characterization of *Sulfurimonas hydrogeniphila* sp. nov., a Novel Bacterium Predominant in Deep-Sea Hydrothermal Vents and Comparative Genomic Analyses of the Genus *Sulfurimonas*

**DOI:** 10.3389/fmicb.2021.626705

**Published:** 2021-02-26

**Authors:** Shasha Wang, Lijing Jiang, Qitao Hu, Liang Cui, Bitong Zhu, Xiaoteng Fu, Qiliang Lai, Zongze Shao, Suping Yang

**Affiliations:** ^1^Department of Bioengineering and Biotechnology, Huaqiao University, Xiamen, China; ^2^Key Laboratory of Marine Genetic Resources, Third Institute of Oceanography, Ministry of Natural Resources, Xiamen, China; ^3^State Key Laboratory Breeding Base of Marine Genetic Resources, Xiamen, China; ^4^Fujian Key Laboratory of Marine Genetic Resources, Xiamen, China; ^5^Southern Marine Science and Engineering Guangdong Laboratory, Zhuhai, China

**Keywords:** *Sulfurimonas hydrogeniphila*, hydrogen oxidation, sulfur oxidation, hydrothermal vent, environmental adaptation

## Abstract

Bacteria of the genus *Sulfurimonas* within the class *Campylobacteria* are predominant in global deep-sea hydrothermal environments and widespread in global oceans. However, only few bacteria of this group have been isolated, and their adaptations for these extreme environments remain poorly understood. Here, we report a novel mesophilic, hydrogen- and sulfur-oxidizing bacterium, strain NW10^T^, isolated from a deep-sea sulfide chimney of Northwest Indian Ocean.16S rRNA gene sequence analysis showed that strain NW10^T^ was most closely related to the vent species *Sulfurimonas paralvinellae* GO25^T^ with 95.8% similarity, but ANI and DDH values between two strains were only 19.20 and 24.70%, respectively, indicating that strain NW10 represents a novel species. Phenotypic characterization showed strain NW10^T^ is an obligate chemolithoautotroph utilizing thiosulfate, sulfide, elemental sulfur, or molecular hydrogen as energy sources, and molecular oxygen, nitrate, or elemental sulfur as electron acceptors. Moreover, hydrogen supported a better growth than reduced sulfur compounds. During thiosulfate oxidation, the strain can produce extracellular sulfur of elemental α-S_8_ with an unknown mechanism. Polyphasic taxonomy results support that strain NW10^T^ represents a novel species of the genus *Sulfurimonas*, and named as *Sulfurimonas hydrogeniphila* sp. nov. Genome analyses revealed its diverse energy metabolisms driving carbon fixation via rTCA cycling, including pathways of sulfur/hydrogen oxidation, coupled oxygen/sulfur respiration and denitrification. Comparative analysis of the 11 available genomes from *Sulfurimonas* species revealed that vent bacteria, compared to marine non-vent strains, possess unique genes encoding Type V Sqr, Group II, and Coo hydrogenase, and are selectively enriched in genes related to signal transduction and inorganic ion transporters. These phenotypic and genotypic features of vent *Sulfurimonas* may explain their thriving in hydrothermal environments and help to understand the ecological role of *Sulfurimonas* bacteria in hydrothermal ecosystems.

## Introduction

Deep-sea hydrothermal vent is one of the most extreme environments on earth and provides unique and diverse habitats for various microorganisms (Zeng et al., 2020). However, the sharp physical and chemical gradients across the vent chimneys and their surroundings impose great challenges to bacterial survival (Sievert et al., 2008a). In the vent ecosystems, biomass production is mainly energized by oxidation of reduced sulfur compounds and hydrogen driving carbon fixation via chemolithoautotrophic microorganisms, which constitute a dominant bacterial group *in situ* (Nakagawa and Takai, 2008).

Among these chemolithoautotrophs, members of the genus *Sulfurimonas* (class *Campylobacteria*) represent one of the most widespread and preponderant mesophilic bacteria in global deep-sea hydrothermal environments. They have been described as strictly chemolithoautotrophic, metabolically versatile sulfur, and/or hydrogen oxidizers and widely distribute in various hydrothermal habitats, including chimneys, sediments, plumes and diffuse-flow vent fluids (Nakagawa et al., 2005; Campbell et al., 2006; Mino et al., 2017; Dick, 2019). For example, the genus *Sulfurimonas* comprised 70 and 20%, respectively, of the bacterial abundance in plume waters and diffuse fluids of deep-sea vents (Akerman et al., 2013; Perner et al., 2013; Han and Perner, 2015). Obviously, bacteria of this genus play the important role in the biogeochemical cycles of hydrothermal vent systems.

The *Sulfurimonas* genus currently contains eight species with validly published names. Only two strains, *Sulfurimonas autotrophica* OK10^T^ and *Sulfurimonas paralvinellae* GO25^T^, were isolated from deep-sea hydrothermal environments, respectively, from sediments and polychaete nests in Mid-Okinawa Trough hydrothermal field (Inagaki et al., 2003; Takai et al., 2006). Four species were isolated from coastal marine sediments, including *Sulfurimonas denitrificans* DSM1251^T^, *Sulfurimonas hongkongensis* AST-10, *Sulfurimonas xiamenensis* 1-1N^T^ and *Sulfurimonas lithotrophica* GYSZ_1^T^ (Timmer-ten, 1975; Cai et al., 2014; Wang et al., 2020b). Another marine isolate representing *Sulfurimonas gotlandica* GD1^T^ was from the pelagic redox cline of the Baltic Sea (Labrenz et al., 2013). In addition, *Sulfurimonas crateris* SN118^T^ was from a terrestrial mud volcano (Ratnikova et al., 2019).

Of the two deep-sea hydrothermal vent strains, only *S. autotrophica* OK10^T^ has genome sequence publicly available (Sikorski et al., 2010). Genomic analysis indicated that strain OK10^T^ possess all genes essential for carbon fixation via the reductive tricarboxylic acid (rTCA) cycle. Oxidation of reduced sulfur compounds by strain OK10^T^ proceeds via the Sox pathway and sulfide: quinone oxidoreductase (Sqr) (Sikorski et al., 2010). Furthermore, metagenomic and metatranscriptomic analyses revealed the processes and activities involving sulfur/hydrogen oxidation, oxygen respiration and denitrification as well as carbon fixation in genus *Sulfurimonas* inhabiting in vent fluids of Axial Seamount (Fortunato and Huber, 2016). These metabolic pathways were also observed in other hydrothermal samples such as actively venting chimney of East Pacific Rise and hydrothermal chimneys from the Roman Ruins vent field based on metagenomic and metaproteomic analyses (Pjevac et al., 2018; Hou et al., 2020).

To define the ecological roles of *Sulfurimonas* in deep-sea hydrothermal environments, culturable bacteria representing the predominant member *in situ* are required. Six potential novel species of genus *Sulfurimonas* were recently isolated from different marine environments, including three from deep-sea hydrothermal vents, one from deep-sea sediment and two from coastal marine sediments (Wang et al., 2020a). Two isolates from coastal environments, *S. xiamenensis* 1-1N^T^ and *S. lithotrophica* GYSZ_1^T^, have been just assigned as novel species (Wang et al., 2020b). Bacteria of novel species, represented by strain NW10, were recently reported to predominate the bacterial population in *in situ* deep-sea hydrothermal vents globally (Wang et al., 2020a). To understand its environment adaptation and ecological role in hydrothermal ecosystems, we firstly characterized it via a polyphasic taxonomic approach as a novel species. Further, comparative genomic analyses were carried out between vent and non-vent marine *Sulfurimonas* to reveal the unique genotypic features that help to clarify their adaptation to deep-sea hydrothermal environments.

## Results

### Enrichment of Chemolithoautotrophic Sulfur-Oxidizing Bacteria (CSOB) and Isolation of *Sulfurimonas* Species

To obtain CSOB from a newly discovered hydrothermal vent on the Carlsberg Ridge, active vent chimney samples were ground on board and inoculated into sealed bottles filled with MMJHS medium with thiosulfate and hydrogen as the energy sources. After about one and half month incubation on board, enriched bacterial cultures were transferred into 10 ml MMJHS medium, and incubated at 28°C in the laboratory. After 2 days of incubation, bacterial growth was obvious with cells in form of short rods. The bacterium was subsequently purified with the dilution-to-extinction method. The culture in the serum bottle showing growth at the highest dilution was designated as strain NW10^T^. The purity of the culture was further confirmed by microscopic examination and 16S rRNA gene sequencing. Interestingly, the bacterium can produce large amount of elemental sulfur in the form of extracellular granules as determined below.

### Morphology of Strain *Sulfurimonas* sp. NW10^T^

Morphological observations by phase-contrast light microscopy and transmission electron microscope showed that cells of strain NW10^T^ were Gram-negative, motile and short rod-shaped with a size of 0.4–0.8 μm wide and 0.8–3.5 μm long (Supplementary Figure S1). Spore formation was not found during the culture incubation. Cells in older cultures tended to form aggregates. These morphological features are shared with other vent species *S. autotrophica* OK10^T^ and *S. paralvinellae* GO25^T^ (Table 1). In addition, on solid MMJHS medium agar plates, strain NW10^T^ formed small, white, round-shaped colonies with smooth boundaries.

**TABLE 1:** Comparison of characteristics of Sulfurimonas hydrogeniphila NW10T sp. nov. with related species of the genus *Sulfurimonas*.

**Characteristic**	**1**	**2**	**3**	**4**	**5**	**6**	**7**	**8**	**9**
**Shape motility**	**Rods+**	**Rods+**	**Rods+**	**Rods to slightly curved –**	**Rods to slightly curved+**	**Rods to spirilla-like –**	**Rods ND**	**Rods+**	**Curved rods to spirilla-like+**
Doubling time under optimal	6	13–16	1.4	12	8	12	6.1	2.2	13
Temperature range (optimal T) (°C)	4–45 (33)	4–35 (30)	10–40 (23–26)	10–45 (30)	4–45 (33)	10–30 (22)	15–35 (30)	5–40 (30)	4–20 (15)
pH range (optimal pH)	5.0–9.0 (6.0–6.5)	5.4–8.6 (6.1)	5.0–9.0 (6.5)	5.5–8.0 (7.0)	5.0–8.5 (6.5)	ND (7)	6.5–8.5 (7.0–7.5)	5.5–9.5 (8.0)	6.5–8.4 (6.7–8.0)
NaCl requirement	+	+	+	–	+	–	+	+	+
Maximum O_2_ concentration (%)	20	10	15	20	20	0.5	ND	Atmospheric oxygen concentration	10 = 20
Energy sources	H_2_, S^0^, S_2_O_3_^2^, ^–^ HS^–^	H_2_,S^0^, S_2_O_3_^2–^	S^0^, S_2_O_3_^2–^	H_2_, S^0^, S_4_O_6_^2–^, S_2_O_3_^2–^, HS^–^	H_2_, S^0^, S_2_O_3_^2–^ HS^–^	HS^–^, S_2_O_3_^2–^	H_2_, HS^–^, S_2_O_3_^2–^	HS ^–^, S^0^, S_2_O_3_^2–^	H_2_, S^0^, HS^–^, S_2_O_3_^2–^
Organic electron donors	–	–	–	–	–	Formate, fumarate, yeast extract alcohol mix	–	–	Formate, acetate, yeast extract, pyruvate, amino acid
Electron acceptors	S^0^, NO_3_^–^, O_2_	NO_3_^–^, O_2_	O_2_	S^0^, NO_3_^–^, O_2_	S^0^, NO_3_^–^, O_2_	NO_3_^–^, NO_2_^–^, O_2_	NO_3_^–^	NO_3_^–^, NO_2_^–^, O_2_	NO_3_^–^, O_2_
Fatty acids (mol%)									
14:0	4.12	5	8.4	1.1	3.2	0.4	4.8	4.6	0.9
3-OH 14:0	6.44	7			4.9				2.5
16:1 ω7c	31.58	22	45.2	31.8	50.5	67.9		53.4	66.0
16:1 ω5c + t	1.33					2.0			1.3
16:0	28.22	25	37.1	23.4	18.9	15.3	32.8	30.8	15.5
18:0	5.03	4		10.02			16.9	0.4	
18:1 ω7c	18.43	37		18.7	12.1	12.1		9.5	13.1
18:1trans			9.4						
G + C content (mol%)	37.3	37.6	35.2	34.5	33.2	36	34.9	38.8	33.6

*Taxa: 1, Sulfurimonas hydrogeniphila sp. nov. NW10^T^; 2, Sulfurimonas paralvinellae GO25^T^; 3, Sulfurimonas autotrophica OK10^T^; 4, Sulfurimonas xiamenensis 1-1N^T^; 5, Sulfurimonas lithotrophicum GYSZ_1^T^; 6, Sulfurimonas denitrificans DSM 1251^T^;7, Sulfurimonas. hongkongensis AST-10^T^; 8, Sulfurimonas crateris SN118^T^; 9, Sulfurimonas gotlandica GD1^T^; ND, Not determined; +, positive;* –, *negative.*

### Phylogenetic Analysis Based on 16S rRNA and Core Gene Sequences

**FIGURE 1:**
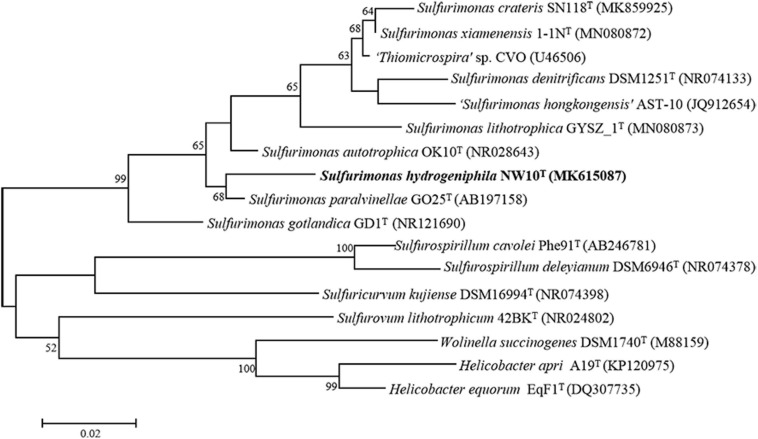
Maximum likelihood phylogenetic tree based on 16S rRNA gene sequences showing the relationship of *Sulfurimonas hydrogeniphila* NW10^T^ with other members within the genus *Sulfurimonas*. Bootstrap values based on 1,000 replicates are shown at branch nodes. Branch node values below 50% are not shown. Bar = 0.02 substitutions per nucleotide position.

**FIGURE 2:**
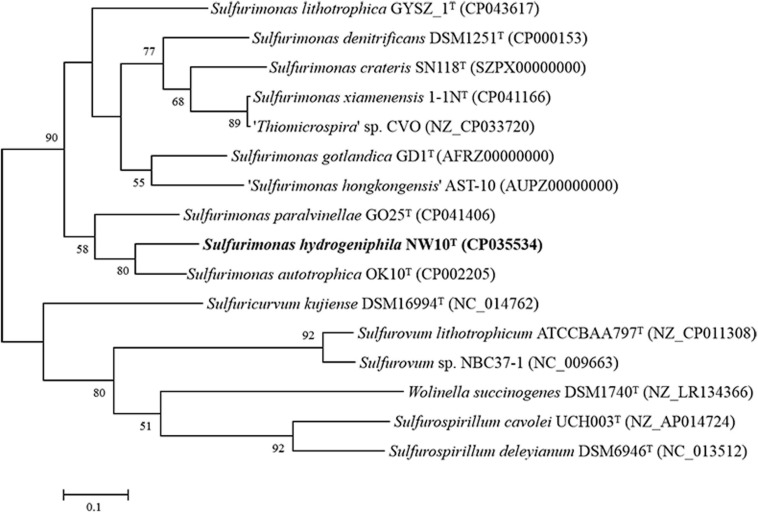
Phylogenetic tree inferred using UBCGs showing the position of *Sulfurimonas hydrogeniphila* NW10^T^ and closely related taxa within the genus *Sulfurimonas* using the maximum-likelihood algorithm. The node is labeled with Gene Support Index (GSI) values. Branch node values below 50% are not shown. The accession numbers of the genomes are shown in parentheses. Bar, 0.1 substitutions per position.

Sequence comparison of the 16S rRNA gene sequences obtained from PCR amplification (1,447 bp) showed that strain NW10^T^ is closest to the only two deep-sea vent bacterial species *S. paralvinellae* GO25^T^ and *S. autotrophica* OK10^T^, with 95.8 and 95.2% sequence identities, respectively. The maximum-likelihood phylogenetic tree based on 16S rRNA gene sequences showed that strain NW10^T^ clusters with *S. paralvinellae* GO25^T^ within the genus *Sulfurimonas* (Figure 1). This was further confirmed through the phylogenomic trees constructed with the neighbor-joining and minimum evolution methods (Supplementary Figures S2, S3). The phylogenomic tree based on the up-to-date bacterial core gene sequences showed that strain NW10^T^ forms a branch with *S. autotrophica* OK10^T^ and *S. paralvinellae* GO25^T^ (Figure 2), supporting further that strain NW10^T^ should belong to the *Sulfurimonas* genus and likely represents a novel species.

### Physiological Characteristics of the Putative Novel Species

Growth experiment showed that strain NW10^T^ could grow in the range of temperatures (4–45°C), salinities (2–4% (w/v) NaCl), pH (5.0–9.0) and oxygen concentrations (1–20%). Strain NW10^T^ also could use nitrate as sole electron acceptor in the absence of oxygen. The optimal growth occurred at 33°C, 6% O_2_, pH 6.0–6.5 and 3% (w/v) NaCl (Table 1). Under optimal growth conditions, the maximum cell concentration was approximately 5.0 × 10^8^ cells/ml and the doubling time was approximately 6 h. Chemoautotrophic growth showed that strain NW10^T^ could grow with thiosulfate, sulfide, elemental sulfur, and hydrogen as energy sources, but not with sulfite and tetrathionate. Strain NW10^T^ grew better with hydrogen as the sole energy source, which brought about the highest cell concentration far more than other energy sources. Thus, hydrogen is possibly the preferred energy source for this bacterium. Similarly, hydrogen also supported the best growth of *S. paralvinellae* GO25^T^ (Takai et al., 2006). When hydrogen was used as the energy source, strain NW10^T^ could respire element sulfur. The product of sulfur reduction was sulfide, which reached up to 42 μM at the late exponential phase in the medium (Wang et al., 2020a). As a chemolithoautotroph, strain NW10^T^ could not grow using any of the tested organic compounds as the carbon source. In addition, none of these organic compounds could be used as an energy source.

The predominant cellular fatty acids of strain NW10^T^ were C_16__:__1_ ω7c (31.6%), C_16__:__0_ (28.2%), C_18__:__1_ ω7c (18.4%) and 3-OH C_14__:__0_ (6.4%), which are similar to those of *S. paralvinellae* GO25^T^, *S. lithotrophicum* GYSZ_1^T^ and *S. gotlandica* GD1^T^, and distinctly different from those of other *Sulfurimonas* species. The significant difference between strain NW10^T^ and its closest relative *S. paralvinellae* GO25^T^ was that its prominent fatty acid in strain NW10^T^ was C_16__:__1_ ω7c, representing 31.6%, whereas in *S. paralvinellae* GO25^T^, C_18__:__1_ ω7c was prominent, accounting for 37.0%. In addition, C_16__:__1_ ω5c + t (1.3%) was observed in strain NW10^T^, but not in *S. paralvinellae* GO25^T^. The fatty acid profile of strain NW10^T^ and the related type strains are detailed in Table 1.

### Generation of Extracellular Biogenic Sulfur and Characterization With SEM, EDS, and Raman Spectroscopy

**FIGURE 3:**
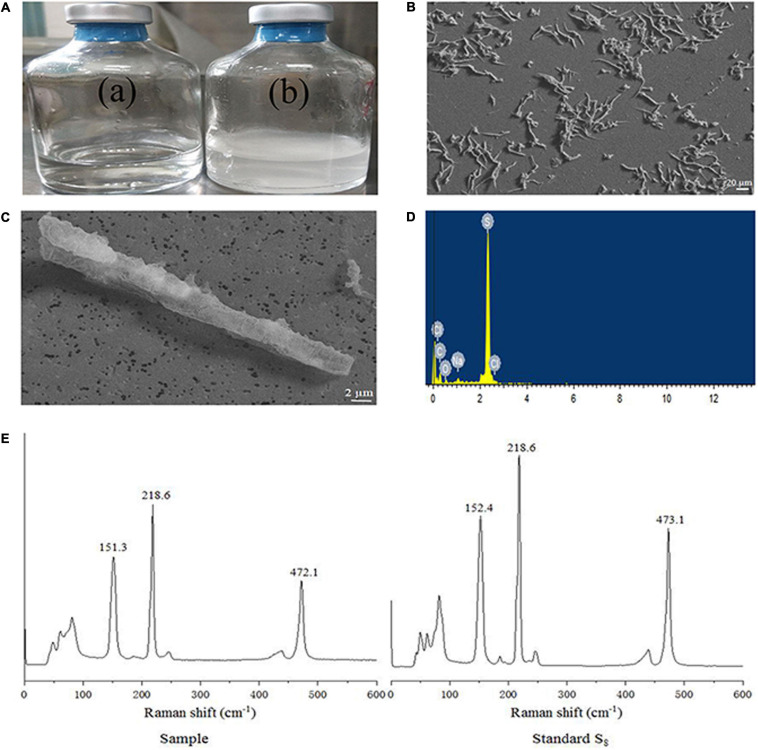
*S. hydrogeniphila* NW10^T^ produces extracellular sulfur when cultured in MMJHS medium. **(A)** Strain NW10^T^ cultured in MMJHS medium without (a) or with 10 mM thiosulfate (b). **(B,C)** SEM observation of S^0^ particles produced by strain NW10^T^. **(D)** Energy dispersive spectrum analysis of extracellular S^0^. **(E)** Raman spectra of extracellular S^0^ produced by strain NW10^T^ and standard S_8._

When strain NW10^T^ was incubated with hydrogen and thiosulfate as mixed electron donors and oxygen as the sole electron acceptor, elemental sulfur occurred in the culture at the mid-exponential growth phase, and accumulated in the late exponential phase and during the stationary phase (Figure 3A). When grown in MMJHS medium with neutral pH such as 7.0 (unbuffered) or weakly acidic pH such as 5.5 (in buffered medium), strain NW10^T^ produced large amount of naked-eye elemental sulfur in the culture. However, there was no accumulation of elemental sulfur observed in alkaline conditions, such as at pH 8.0. Elemental sulfur production was also affected by the oxygen concentration in the gaseous phase and the result showed the maximum accumulation of elemental sulfur occurred at 6% oxygen concentration. Morphological observations under SEM showed that extracellular sulfur was mainly shaped as crystalline bars (Figures 3B,C), with length varying from 10 to 100 μm. EDS analysis revealed that these crystals mainly contained elemental sulfur (Figure 3D). Raman spectroscopy further showed the presence of α-S_8_ when compared against a standard chemical product of S_8_^0^ (Figure 3E). This confirmed that the extracellular aggregates produced by strain NW10^T^ were biogenic sulfur and composed of crystalline α-S_8_. These findings suggested that strain NW10^T^ performs incomplete oxidation of thiosulfate with elemental sulfur as an oxidation intermediate accumulated outside of the cells.

### Genomic Properties and Genetic Relatedness

Strain NW10^T^ has a single circular chromosome (Supplementary Figure S4) with complete genome size of 2,432,011 bp with GC content of 37.3%, which is similar to that of *S. paralvinellae* GO25^T^ (Table 1). No plasmid was detected in the genome of this bacterium. Total 2,472 genes were predicted, which contained 2,367 protein coding genes and 57 RNA genes. The RNA genes cover 45 tRNAs and 12 rRNAs. ANI and DDH were calculated to identify the genomic similarities of strain NW10^T^ with other species of the genus *Sulfurimonas*. Pairwise ANI values between strain NW10^T^ and its closest relatives, *S. paralvinellae* GO25^T^ and *S. autotrophica* OK10^T^, were 74.50 and 81.15%, respectively. The predicted DDH value between strain NW10^T^ and *S. paralvinellae* GO25^T^ was 19.20% and the value between strain NW10^T^ and *S. autotrophica* OK10^T^ was 24.70%. These findings supported the classification of strain NW10^T^ as one distinct species according to the cut-off thresholds of ANI (95–96%) and DDH (70%) for delineation of prokaryotic species (Chun et al., 2018).

### Comparative Genomic Analysis of the *Sulfurimonas* Genus

**FIGURE 4:**
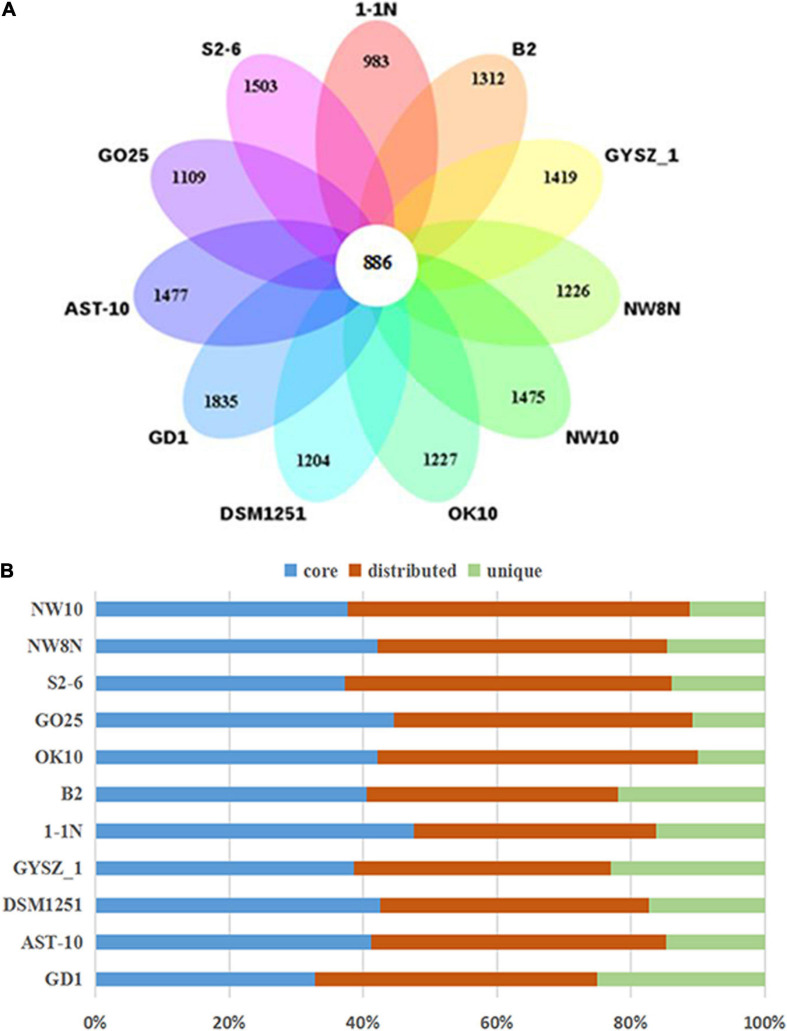
Comparisons of *Sulfurimonas* orthologous protein groups in 11 *Sulfurimonas* genomes. **(A)** Venn diagram displaying the numbers of core gene families and flexible genes for each of the 11 *Sulfurimonas* strains. **(B)** Percentage of core, distributed, and unique genes in each of the 11 genomes.

Pan-genomic studies were carried out to investigate the genotypic features of 11 *Sulfurimonas* species, including five vent bacteria-*S. hydrogeniphila* NW10^T^, *Sulfurimonas* sp. NW8N, *Sulfurimonas* sp. S2-6, *S. paralvinellae* GO25^T^ and *S. autotrophica* OK10^T^, and six non-vent marine bacteria-*Sulfurimonas* sp. B2, *S. xiamenensis* 1-1N^T^, *S. lithotrophica* GYSZ_1^T^, *S. denitrificans* DSM1251^T^, *S. hongkongensis* AST-10 and *S. gotlandica* GD1^T^ (Table 2). Comparative analyses based on orthologous groups of proteins revealed 886 core genes shared by all the eleven *Sulfurimonas* genomes (Figure 4A). The percentages of core genes in each genome ranged from 32.7 to 47.6%, indicating that the 11 strains of *Sulfurimonas* shared a low percentage of common functional proteins. The percentages of unique genes in each genome varied from 10.8 to 23.0%, however, a relative high proportion of distributed genes in each genome was detected, ranging from 36.2 to 51.0% (Figure 4B). The distributed genes usually offer species diversity, environmental adaptation and other characteristics for bacteria (Innamorati et al., 2020). In addition, considering that the definition of unique genes is subject to change, a singleton today may be reclassified into a multi-gene cluster when new genome data is included in the future (Zhang and Sievert, 2014), we decided to focus on the distributed genes to investigate common adaptation characteristics of the *Sulfurimonas* genus to deep-sea hydrothermal vent environments.

**TABLE 2:** General genome features of *Sulfurimonas* species used in this study.

**Strain**	**Genome size (Mb)**	**Chromosomes (plasmid)**	**G + C (%)**	**CDS**	**tRNA**	**Source of Isolation**	**NCBI accession No.**
*Sulfurimona*s *hydrogeniphila* NW10^T^	2.34	1 (0)	37.3	2,473	44	Hydrothermal vent chimneys	CPO35534
*Sulfurimona*s sp. NW8N	2.09	1 (0)	36.8	2,134	36	Hydrothermal vent chimneys	SDID00000000
*Sulfurimona*s sp. S2-6	2.32	1 (0)	37.3	2,421	43	Hydrothermal vent sediments	CP041235
*S. paralvinellae* GO25^T^	2.03	1 (1)	38.6	2,026	41	Hydrothermal vent polychaete nest	CP041406 CP041407
*S. autotrophica* OK10^T^	2.15	1 (0)	35.2	2,140	43	Hydrothermal vent sediments	CP002205.1
*Sulfurimona*s sp. B2	2.26	1 (0)	35.9	2,266	45	Deep-sea sediments	CP041165
*Sulfurimona*s *xiamenensis*1-1N^T^	1.92	1 (0)	34.5	1,891	43	Coastal marine sediments	CP041166
*Sulfurimona*s *lithotrophica* GYSZ_1^T^	2.38	1 (1)	33.2	2,348	41	Coastal marine sediments	CP043617 CP043618
*S. denitrificans* DSM1251^T^	2.20	1 (0)	34.5	2,133	44	Coastal marine sediments	CP000153.1
*S. hongkongensis* AST-10	2.30	1 (0)	34.9	2,290	39	Coastal marine sediments	AUPZ00000000.1
*S*. *gotlandica* GD1^T^	2.95	1 (0)	33.6	2,819	47	Marine sulfidic water of a pelagic redox zone	AFRZ00000000.1

### Central Metabolisms Within Genus *Sulfurimonas*

#### Sulfur Metabolism

**FIGURE 5:**
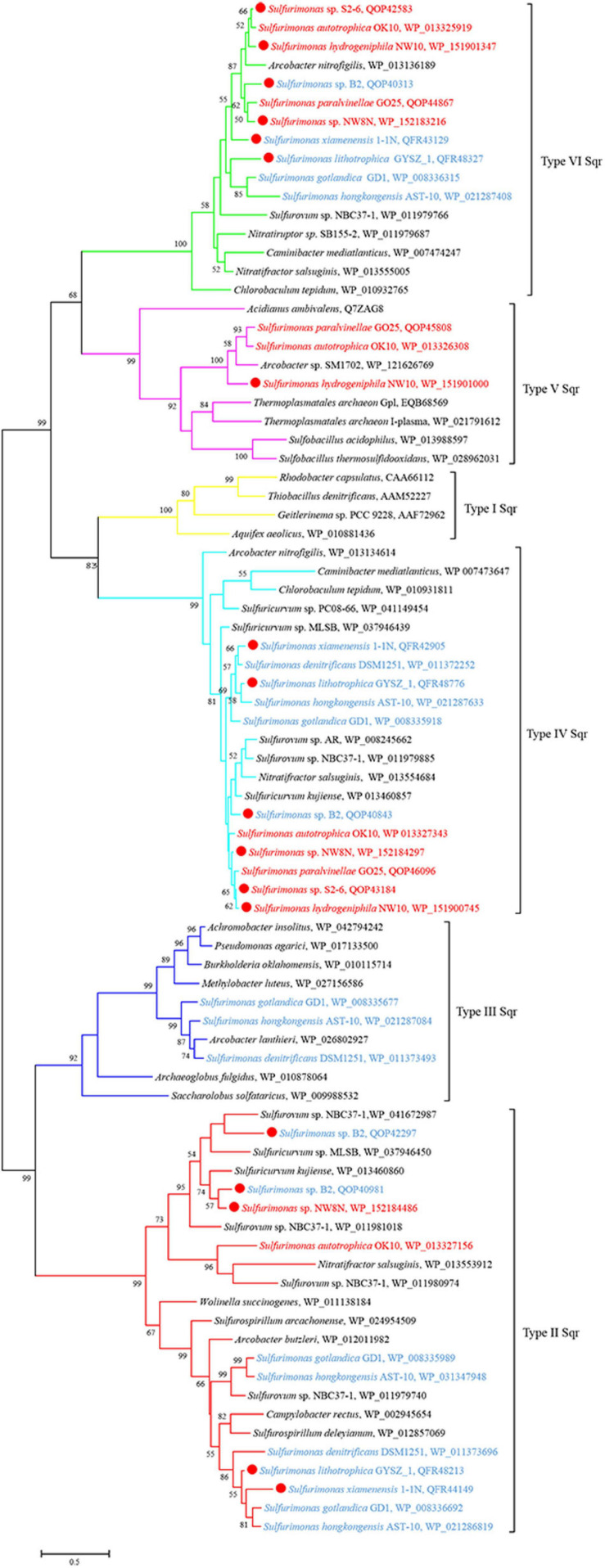
Maximum likelihood phylogenetic tree of Sqr protein sequences derived from *Sulfurimonas* strains and other representative species within the class *Campylobacteria*. Bootstrap values indicated at each node are based on a total of 1,000 bootstrap replicates. Branch node values below 50% are not shown. The solid red circles represent our own isolated strains. Branches representing different type of Sqr are different color. Isolation sources of *Sulfurimonas* species are indicated in different font colors: red, hydrothermal environments; blue, marine non-vent system.

Genomic analysis revealed that strain NW10^T^ contains two Sox gene clusters (*soxABXY_1_Z_1_* and *soxCDY_2_Z_2_*) forming a complete Sox multi-enzyme system (Table 3 and Supplementary Table S1). Furthermore, comparative analysis showed that *soxCDY_2_Z_2_* is present in all *Sulfurimonas* species, indicating that it was conserved among *Sulfurimonas* spp. The gene cluster *SoxABXY_1_Z_1_* was absent in the non-vent strains *S. xiamenensis* 1-1N^T^ and *S. lithotrophica* GYSZ-1^T^. Despite this absence, growth experiments showed that both strains still oxidize thiosulfate to sulfur. Strain NW10^T^ contained three homologs of *sqr* gene, usually responsible for oxidizing sulfide to elemental sulfur. Comparative analysis showed that *Sulfurimonas* species harbor diverse types of Sqr, including Type II, III, IV, V, and VI (Table 3 and Figure 5). As shown in Table 3, except for Type VI Sqr that was absent in *S. denitrificans* DSM1251^T^, both Type IV and VI Sqrs were conserved among all 11 sequenced *Sulfurimonas* species. Phylogenetic analysis showed that Type IV Sqr of hydrothermal vent *Sulfurimonas* clustered together and those from non-vent *Sulfurimonas* species formed another cluster, indicating a differential evolution of Type IV Sqr was in accordance to the environmental origins of the hosts (Figure 5). All non-vent strains harbored Type II Sqr, and among them strains B2, *S. hongkongensis* AST-10 and *S. gotlandica* GD1^T^ had two copies. In contrast, strains NW8N and *S. autotrophica* OK10^T^ from hydrothermal vents only had one copy of type II Sqr. Interestingly, Type III Sqr only occurred in non-vent strains, *S. denitrificans* DSM1251^T^, *S. hongkongensis* AST-10 and *S. gotlandica* GD1^T^. However, Type V Sqr only presented in the three vent strains NW10^T^, *S. autotrophica* OK10^T^ and *S. paralvinellae* GO25^T^. The Type III Sqrs were phylogenetically close to the cluster of Type II (Figure 5), indicating that they may have similar functions, as previously reported by Han and Perner (2015). Phylogenetic analysis also showed that Type V Sqr from strains NW10^T^, *S. autotrophica* OK10^T^ and *S. paralvinellae* GO25^T^ clustered with those of thermophilic bacteria and archaea as well as green sulfur bacteria (Figure 5).

**TABLE 3:** Comparison of key enzymes for sulfur, nitrogen, hydrogen, and carbon metabolisms in *Sulfurimonas* species based on RAST annotations in this study.

	**NW10N**	**S2-6**	**NW8N**	**OK10**	**GO25**	**B2**	**1-1N**	**GYSZ_1**	**GD1**	**AST-10**	**DSM1251**
**Sulfur metabolisms**											
Sulfur oxidation protein (Sox)	SoxABXYZ; SoxCDYZ	SoxABXYZ; SoxCDYZ	SoxABXYZ; SoxCDYZ	SoxABXYZ; SoxCDYZ	SoxABXYZ; SoxCDYZ	SoxABXYZ; SoxCDYZ	SoxCDYZ	SoxCDYZ	SoxABXYZ; SoxCDYZ	SoxABXYZ; SoxCDYZ	SoxABXYZ; SoxCDYZ
Sulfide:quinone oxidoreductase (Sqr)	IV; V; VI	IV; VI	II; IV; VI	II; IV; V; VI	IV; V; VI	II (2); IV; VI	II; IV; VI	II; IV; VI	II (2); III; IV; VI	II (2);III;IV;VI	II; III; IV
Sulfite dehydrogenase (Sor)	–	+	+	+	+	+	–	+	+	+	–
Assimilatory sulfur reduction	Sat; CysC; CysDN	Sat; CysC; CysDN	Sat; CysC; CysDN	Sat; CysC; CysDN	Sat	Sat	Sat	Sat; CysC; CysDN	Sat;CysC (2); CysDN (2); CysH; CysI	Sat	Sat; CysC; CysDN; CysI
**Hydrogen metabolism**											
[NiFe]-Hydrogenases Group 1	+	+ (2)	+ (2)	+	–	+	(2)	+ (2)	+ (3)	+ (2)	+
[NiFe[-Hydrogenases Group 2	+	+	+	–	–	–	+	+	+	+	+
[NiFe[-Hydrogenases Group 4	–	+	–	+	+	–	–	–	+	–	–
**Nitrogen metabolisms**											
Periplasmic nitrate reductase (Nap)	+	+	+	+	+	–	+	+	+	+	+
Nitrate assimilation	+	+	+	+	+	–	–	+	+	–	–
**Carbon metabolisms**											
Oxoglutarate:ferredoxin oxidoreductase (Oor)	+	+	+	+	+	+	+	+	+	+	+
Pyruvate:ferredoxin oxidoreductase (Por)	+	+	+	+	+	+	+	+	+	+	+
ATP-dependent citrate lyase (Acl)	+	+	+	+	+	+	+	+	+	+	+

*+Present; the number of enzymes are listed in the brackets; –not present.*

Besides Sox multienzyme complex and Sqr, *sorAB* genes responsible for sulfite oxidation were detected in most *Sulfurimonas* genomes, but absent in strains NW10^T^, 1-1N^T^ and *S. denitrificans* DSM1251^T^ (Table 3). In addition, strain NW10^T^ had an incomplete assimilation sulfate reduction pathway, only containing genes encoding sulfate adenylyltransferase (*Sat*), adenylylsulfate kinase (*CysC*) and sulfate adenylyltransferase (*CysDN*), which were also found in all other *Sulfurimonas* bacteria except strain *S. gotlandica* GD1^T^ (Table 3).

#### Hydrogen Metabolism

**FIGURE 6:**
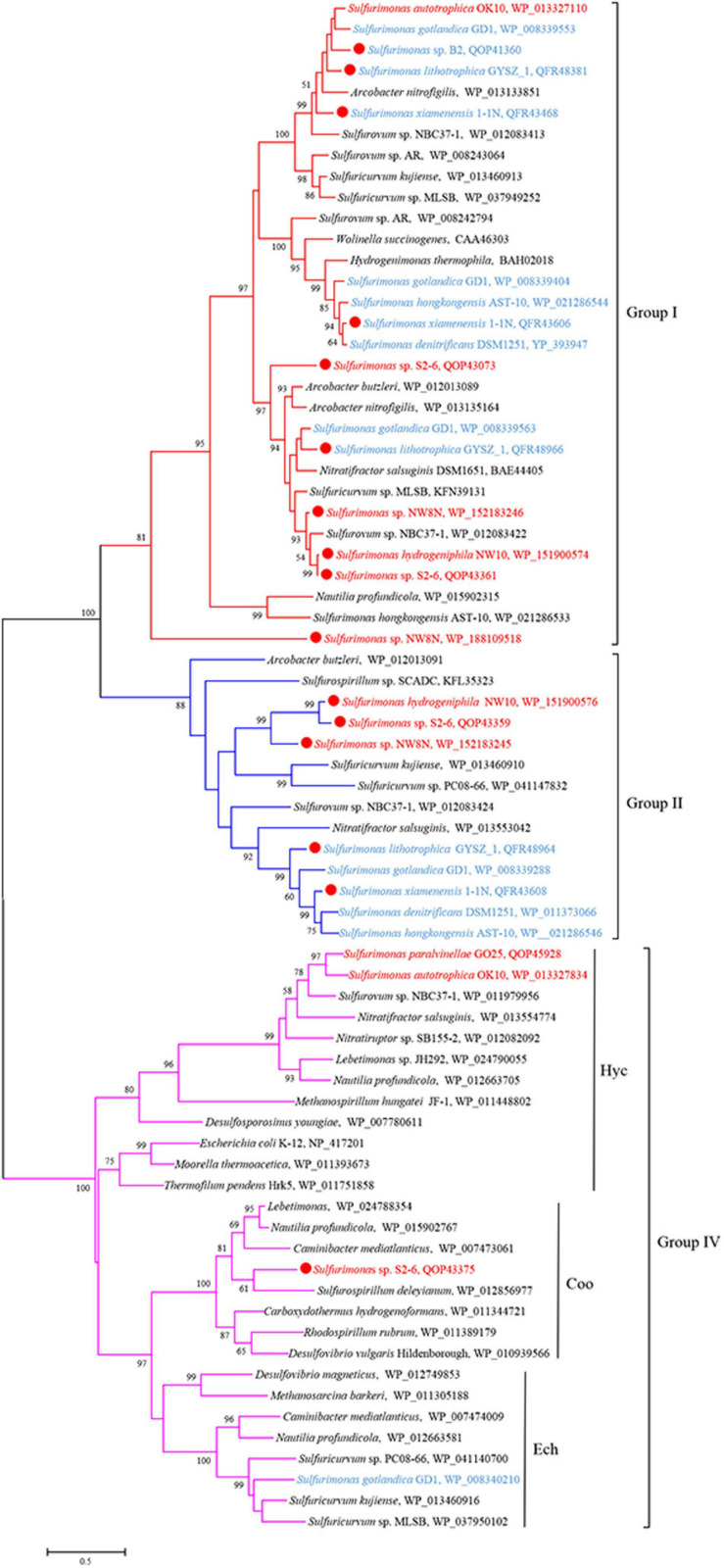
Maximum likelihood phylogenetic tree of hydrogenase large subunit sequences derived from *Sulfurimonas* strains and other representative species within the class *Campylobacteria*. Bootstrap values indicated at each node are based on a total of 1,000 bootstrap replicates. Branch node values below 50% are not shown. The solid red circles represent our own isolated strains. Branches representing different hydrogenase group are different color. Isolation sources of *Sulfurimonas* species are indicated in different font colors: red, hydrothermal environments; blue, marine non-vent system.

Genomic analysis revealed that *Sulfurimonas* spp. contain three types of hydrogenases: [NiFe]-Hydrogenases Group 1 (Hyd and Hyp), Group 2b (Hup) and Group 4 (Hyc, Coo, and Ech) (Table 3). Except for *S. paralvinellae* GO25^T^, all *Sulfurimonas* species contained the Group 1 hydrogenases (Table 3), suggesting that this group might be essential for growth. Strains S2-6, NW8N, 1-1N^T^, GYSZ_1^T^, and *S. hongkongensis* AST-10 had two Group I hydrogenases, and strain *S. gotlandica* GD1^T^ had three. Phylogenetic analysis showed that Group I hydrogenases of *Sulfurimonas* grouped into different clusters with diverse *Campylobacteria* (Figure 6). Group II hydrogenases existed in most of *Sulfurimonas* species (Table 3) and surprisingly, three vent strains, i.e., NW10^T^, NW8N, and S2-6, also harbored this type of hydrogenase. It is the first observation that Group II hydrogenases are present in vent *Sulfurimonas* species. Type IV hydrogenases were only found in four *Sulfurimonas* species, strains S2-6, *S. autotrophica* OK10^T^, *S. paralvinellae* GO25^T^, and *S. gotlandica* GD1^T^ (Table 3). Phylogenetic analysis showed that group IV hydrogenase can be further classified into three types: Hyc, Coo and Ech. Hyc proteins from *S. autotrophica* OK10^T^ and *S. paralvinellae* GO25^T^ were clustered with those from other vent *Campylobacteria* (Figure 6). Ech was only found in strain *S. gotlandica* GD1^T^, and Coo was only found in vent strain S2–6.

#### Nitrogen Metabolism

**FIGURE 7:**
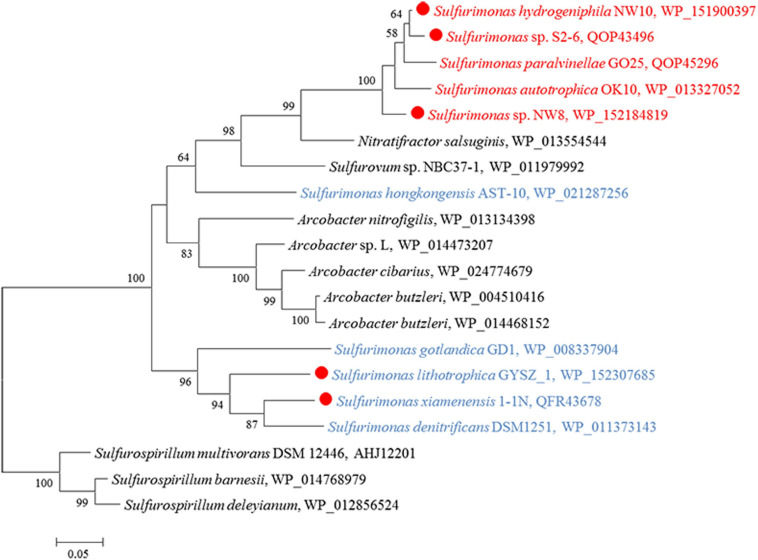
Maximum likelihood phylogenetic tree of the NapA protein sequences derived from *Sulfurimonas* strains and other representative species within the class *Campylobacteria*. Bootstrap values indicated at each node are based on a total of 1,000 bootstrap replicates. Branch node values below 50% are not shown. The solid red circles represent our own isolated strains. Isolation sources of *Sulfurimonas* species are indicated in different font colors: red, hydrothermal environments; blue, marine non-vent system.

Except for strain B2, genes encoding all components required for the complete reduction of nitrate to nitrogen gas, i.e., nitrate reductases (*nap*), nitrite reductases (*nir*), nitric oxide reductases (*nor*) and nitrous oxide reductases (*nos*), were found in all *Sulfurimonas* species (Table 3). However, even though the genomes of strains NW8N and *S. autotrophica* OK10^T^ contained the *napAGHBFLD* operon, both strains were incapable of growing with nitrate as the sole electron acceptor under the tested conditions. We speculated that strains NW8N and *S. autotrophica* OK10^T^ may use nitrate as the electron acceptor under certain unidentified environmental conditions. Phylogenetic analysis showed that NapAs from vent *Sulfurimonas* species clustered together, while those from non-vent but marine habitats clustered apart and were close to those from *Arcobacter* species (Figure 7). In addition, strain NW10^T^ had the complete assimilatory nitrate reduction pathway, containing the genes encoded nitrate transporter (*nasAB*) and ferredoxin-nitrite reductase (*nirA*). This gene cluster was also found in all other hydrothermal vent isolates, but absent in most non-vent strains (4/6) (Table 3).

#### Central Carbon Metabolism

**FIGURE 8:**
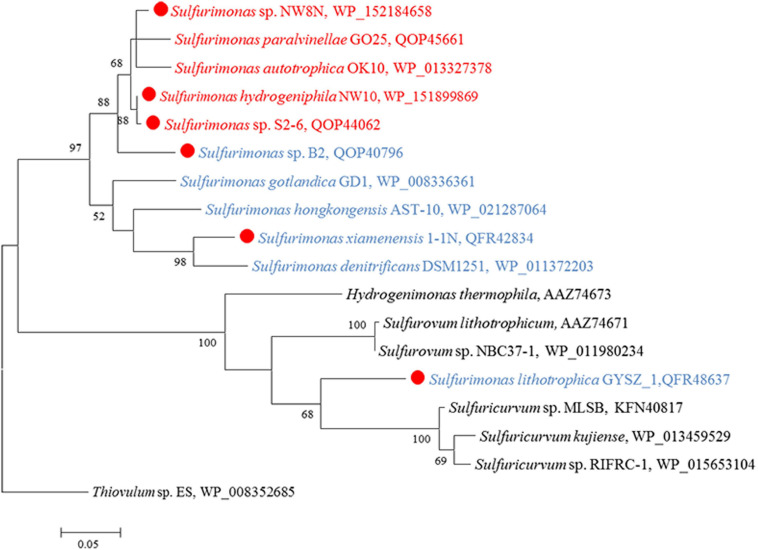
Maximum likelihood phylogenetic tree of the AclB protein sequences derived from *Sulfurimonas* strains and other representative species within the class *Campylobacteria*. Bootstrap values indicated at each node are based on a total of 1,000 bootstrap replicates. Branch node values below 50% are not shown. The solid red circles represent our own isolated strains. Isolation sources of *Sulfurimonas* species are indicated in different font colors: red, hydrothermal environments; blue, marine non-vent system.

CO_2_ fixation coupled to oxidation of reduced sulfur compounds and hydrogen is a typical characteristic in *Campylobacterial* species for biomass production (Waite et al., 2017). Strain NW10^T^ was capable of chemoautotrophic growth with CO_2_/HCO_3_^–^. All of the enzymes essential for rTCA cycle were encoded in strain NW10^T^, including ATP-dependent citrate lyase (Acl), 2-oxoglutarate: ferredoxin oxidoreductase (Oor) and pyruvate:ferredoxin oxidoreductase (Por) (Table 3). These key enzymes for CO_2_ fixation were also found in all other *Sulfurimonas* species (Table 3). Phylogenetic analysis showed that the AclBs from *Sulfurimonas* species formed a separate clade, with the exception of strain GYSZ_1^T^ (Figure 8), suggesting this pathway is conserved in *Sulfurimonas* species. In addition, AclBs from vent isolates clustered together, while non-vent-AclBs formed a distinct cluster.

### Niche-Specific Genes at the Deep-Sea Hydrothermal Vents

The niche-specific genes were retrieved based on comparative genome analysis. We compared the genes common in five hydrothermal vent strains with those shared by non-hydrothermal vent strains. Overall, 57 unique genes specific of the hydrothermal vent isolates were found (Supplementary Table S2). The hydrothermal vent-specific genes encoded four major functions, including signal transduction, energy production and conversion, inorganic ion transport and cell wall/membrane/envelope biogenesis (Supplementary Table S2).

As for signal transduction, some transcriptional regulators belonging to the Per-ARNT-Sim (PAS) family were shared by the hydrothermal vent strains (Supplementary Table S2). Two histidine kinases (clusters 1,373 and 1,406), as the sensing component of the two-component signal transduction system, and one response regulator (cluster 1,293) were also found across the five vent strains (Supplementary Table S2), which may be responsible for sensing certain hydrothermal vent environmental conditions. In addition, the genomes of vent strains encoded relatively high numbers of signaling proteins (Supplementary Table S2), and particularly the genes encoding proteins with EAL and GGDEF domains, likely involved in the synthesis and hydrolysis of the intracellular signaling compound cyclic diguanylate. Regarding energy conservation, some genes involved in energy metabolism such as pyruvate dehydrogenase complex, which converts pyruvate to acetyl-CoA, NADH and CO_2_, were also shared by vent strains (Supplementary Table S2). We also found three rhodanese-related sulfur transferase (clusters 1,452, 1,501, and 1,507), involved in sulfur metabolism, uniquely present in the genomes of all vent strains. In addition, the vent strains shared multiple transporters, including the ABC transporter systems (clusters 1,349, 1,362, and 1,399) and some metal ion transporters such as Zn^2+^, Mg^2+^, Cu^2+^ and K^+^ (Supplementary Table S2). Finally, we found three outer membrane proteins TolC (clusters 1,471, 1,475, and 1,511), previously associated with different efflux systems and type I protein secretion (Pérez-Llarena and Bou, 2016), shared by all vent strains.

## Discussion

Chemoautotrophic bacteria of the genus *Sulfurimonas* in the class *Campylobacteria* are ubiquitous and numerically dominant in global deep-sea hydrothermal vents (Mino et al., 2017; Dick, 2019). It is clear that *Sulfurimonas* species likely play an important role in the biogeochemical cycles of carbon, nitrogen and sulfur. However, yet little is known about their adaptations to vent environments. In this study, a novel species designated strain NW10^T^ was characterized, which was isolated from a sulfide chimney on the Carlsberg Ridge of Northwestern Indian Ocean. In addition, we carried out a comparative genomic analysis including core and distributed genes to gain insights into the adaptation mechanisms of *Sulfurimonas* species to the deep-sea hydrothermal vents.

### A New Species of *Sulfurimonas* Genus Representing a Predominant Bacterium *in situ*

Strain NW10^T^ shared the highest 16S rRNA gene sequence similarity (95.8%) with *S. paralvinellae* GO25^T^ and formed a phylogenetic subcluster within the genus *Sulfurimonas*, indicating that this strain should belong to the *Sulfurimonas* genus. Furthermore, ANI and DDH analyses clearly showed that strain NW10^T^ could be differentiated genetically from other previously described *Sulfurimonas* species. Phenotypically, strain NW10^T^ was different from its closest relative *S. paralvinellae* GO25^T^ in many characteristics (Table 1), such as the growth conditions and maximum growth rate. The utilization patterns of electron donor were also different, in that strain NW10^T^ could oxidize sulfide and elemental sulfur, whereas *S. paralvinellae* GO25^T^ could not (Table 1). The combined evidence in phenotypic, chemotaxonomic and phylogenetic features conclusively demonstrates that strain NW10^T^ represents a novel species in the genus *Sulfurimonas*, for which the name *Sulfurimonas hydrogeniphila* sp. nov. is proposed.

In deep-sea hydrothermal environments, bacteria of this species are widely spread, as supported by ecological distribution search in different environments through the Integrated Microbial Next Generation Sequencing (IMNGS) (Lagkouvardos et al., 2016). Across the 274,621 deep-sea hydrothermal vent samples gathered in the IMNGS platform, the relative abundance of *Sulfurimonas* sp. NW10-like sequences (>97% similarity of 16S rRNA gene in length 1,447 bps) was more than 1% total sequence reads in 49% of the vent samples and more than 0.1–1% in 39% of the vent samples (Wang et al., 2020a). The highest relative abundance was found in a deep-sea hydrothermal vent fluid sample from the Pacific Ocean, accounting for 22.4% of the total 16S sequences (Wang et al., 2020a). The relative high abundance and wide distribution of the new species highlight its importance in the biogeochemical cycles of sulfur in *in situ* hydrothermal environments.

### Incomplete Thiosulfate Oxidation and Extracellular Biogenic S^0^

In sulfur-oxidizing bacteria, thiosulfate is usually oxidized by a Sox multi-enzyme system (SoxABCDXYZ) located in the periplasm. Two kinds of Sox pathways have been described according to the presence or absence of SoxCD. When SoxCD is present, it acts as a sulfur dehydrogenase and completely oxidizes thiosulfate to sulfate without formation of sulfur globule. Otherwise, sulfur is formed as an intermediate without SoxCD (Frigaard and Dahl, 2008). In this study, we found the *soxCD* genes were present in all *Sulfurimonas* genomes (Table 3). In addition, previous study showed that *Sulfurimonas* species did perform a complete thiosulfate oxidation without elemental sulfur as an intermediate (Inagaki et al., 2003; Sievert et al., 2008a; Labrenz et al., 2013). However, strain NW10^T^ seems to be an exception in genus *Sulfurimonas*. Despite the presence of SoxCD, it performed incomplete oxidation of thiosulfate, with elemental sulfur accumulation outside of the cells (Figure 3).

The accumulation of extracellular S^0^ was significantly influenced by culture conditions, such as pH. At alkaline pH, strain NW10^T^ completely oxidized thiosulfate to sulfate, while under neutral and acidic conditions, thiosulfate was oxidized to sulfate, with formation of elemental sulfur as an intermediate. The phenomenon was also observed in different strains of *Hydrogenovibrio* genus (Javor et al., 1990; Houghton et al., 2016; Jiang et al., 2017). Considering that the vent fluids of black chimneys are typically acidic, it is likely that the *Sulfurimonas* species inhabiting on vent chimneys can generate extracellular S^0^ *in situ*. In addition, oxygen concentrations can also significantly influence the elemental sulfur generation, with a maximum accumulation of extracellular S^0^ occurred at 6% oxygen.

Furthermore, the structures of extracellular S^0^ produced by NW10^T^ were mainly in the form of crystalline bars with clear edges composed of α-S_8_, which was significantly different from the sulfur globules usually resulting from most chemotrophic and phototrophic bacteria activity (Dahl and Prange, 2006; Jiang et al., 2017; Cron et al., 2019). α-S_8_ is the thermodynamically most stable form of elemental sulfur at ambient temperature and pressure, and has been found in very diverse environments, such as marine sediments, water columns, euxinic lakes, sulfidic caves, hydrothermal vents, as well as cold or hot springs (Roy and Trudinger, 1970; Taylor et al., 1999; Steudel, 2000; Gleeson et al., 2012; Findlay et al., 2014; Hamilton et al., 2015). This crystalline sulfur could serve as an important intermediate in the biogeochemical sulfur cycle, and be further consumed by a wide diversity of microorganisms, such as sulfur oxidizer, sulfur reducer, or sulfur disproportionator. Our results highlight the potential ecological significance of extracellular S^0^ produced by *Sulfurimonas* in deep-sea hydrothermal ecosystems.

The mechanisms involved in the formation of extracellular S^0^ by SOB are still enigmatic compared with those of intracellular S^0^, although a variety of chemotrophic and phototrophic SOB and archaea can produce extracellular S^0^ (Dahl and Friedrich, 2008). So far, a large number of studies focused on the analysis of chemical form and structure of microbial extra-and intra-cellular S^0^ (Pickering et al., 2001; George et al., 2008; Berg et al., 2014). To our knowledge, the enzymes catalyzing the oxidation of sulfide or thiosulfate to S^0^ are usually located in the bacterial periplasm, but sulfur globules are observed outside of the cells, and sometimes even keeping the cells at a distance (Gregersen et al., 2011; Marnocha et al., 2016; Cron et al., 2019). Therefore, it has been proposed that reduced sulfur compounds could be initially oxidized to soluble polysulfide intermediates in the periplasm, and then be transported outside the cells to form sulfur globules. It is still not clear how extracellular S^0^ can accumulate outside of the cells (Cron et al., 2019). Recently, increasing number of studies indicate that SOB could excrete soluble organics to help form and stabilize S^0^ in the environment (Cosmidis et al., 2019; Cron et al., 2019; Marnocha et al., 2019). This could explain many of the puzzling fact observed in microbial extracellular S^0^, such as coated by organics on the surface, and growing extracellularly at a distance from the cells (Hanson et al., 2016; Marnocha et al., 2016, 2019). Here, the extracellular S^0^ produced by strain NW10^T^ mainly contained elemental sulfur, with extremely low amounts of carbon and oxygen. In addition, there were no known homologs of sulfur globule proteins (SGP) genes in its genome, and comparative genome analysis showed no significant difference in Sox pathways between strain NW10^T^ and other *Sulfurimonas* species and the only difference is that SoxC protein of strain NW10^T^ lacks 18 bases at the N-terminal. The mechanism of extracellular S^0^ production requires further investigations by means of multiple omics in future.

### Environmental Adaptations of *Sulfurimonas* Revealed by Comparative Genomic Analyses

Bacteria of the *Sulfurimonas* genus have been found to colonize a broad range of natural habitats from terrestrial, coastal sediment, shallow waters to deep-sea hydrothermal vents globally (Grote et al., 2008; Dahle et al., 2013; Meier et al., 2017). In this report to understand their environmental adaptation, we carried out comparative genomic analyses between vent and non-vent marine *Sulfurimonas* strains. Regarding energy conservation, all *Sulfurimonas* genomes contained the broad suite of genes encoding the enzymes capable of oxidizing thiosulfate, sulfite, sulfide and hydrogen (Table 3). In relation to sulfide oxidation, *Sulfurimonas* species contained genes encoding diverse types of Sqrs, allocated into Types II, III, IV, V, and VI. The variation and diversification of these Sqrs are presumed to play important roles in sulfide oxidation, sulfide assimilation, energy generation, heavy metal tolerance, detoxification and sulfide signaling (Marcia et al., 2010). Type IV and VI Sqrs were relatively conserved in genus *Sulfurimonas* (Table 3), likely to cope with different concentrations of sulfide, such as free sulfide in hydrothermal vents and metal sulfide in marine sediments (Han and Perner, 2015). Additionally, *Sulfurimonas* bacteria had one or more copies of Type II Sqrs (Table 3), which may compensate for the function of other Sqrs under specific environmental conditions (Han and Perner, 2015). Distinct roles of Type II Sqr have been proposed in different microorganisms. For example, it may be involved in heavy metal tolerance in the yeast *Saccharomyces pombe* (Vande-Weghe and Ow, 1999), sulfur assimilation in the non-pathogenic bacterium *Pseudomonas putida* KT2440 (Shibata and Kobayashi, 2006), and sulfide signaling in mammalian cells (Shahak and Hauska, 2008). In addition, some *Sulfurimonas* species harbored Type III and V Sqrs besides Type II, IV and VI Sqrs (Table 3), which may function in sulfide oxidation to enhance energy generation or detoxification and sulfide signaling (Han and Perner, 2015). It is interesting to find that Type V Sqr is only present in vent strains, such as NW10^T^, S2-6 and *S. autotrophica* OK10^T^. Type V Sqrs are a group of archaeal proteins, predominantly found in *Creanarcheaota* and *Euryarchaeota* (Sousa et al., 2018). It was only characterized in the archaeon *Acidianus ambivalens* and has been confirmed to possess Sqr activity with high substrate affinity (Brito et al., 2009). In addition, this type of Sqr in *A. ambivalens* exhibited the highest activity at 70°C, and only 3% activity remained at 25°C (Brito et al., 2009). This indicates that Type V Sqr may tolerate elevated temperatures. Phylogenetic relationship showed that *Sulfurimonas* has likely acquired its Type V Sqr gene from archaea through horizontal gene transfer (Figure 5; Han and Perner, 2015). Further experiments will be required to confirm the temperature tolerance attributed to Type V Sqr. In addition, genes encoding rhodanese-related sulfurtransferase were significantly enriched in vent strains (Supplementary Table S1). Previous study has indicated that the rhodanese-related sulfurtransferase serves as a polysulfide-sulfur transferase at lower polysulfide concentration in *Wolinella succinogenes* (Klimmek et al., 1991) and was possibly involved in polysulfide reduction in *Desulfurella amilsii* (Florentino et al., 2019). Here, we hypothesize that these sulfur transferases from vent strains may play a key role in the sulfur/polysulfide respiration process, where elemental sulfur or polysulfides are abundant.

Hydrogen oxidation is popular in bacteria of the *Sulfurimonas* genus. Members of this genus harbor a relatively broad suite of hydrogenases (Table 3), which can catalyze the hydrogen oxidation coupled to various electron acceptors reduction, including oxygen, nitrate and elemental sulfur. Almost all *Sulfurimonas* members contained multiple copies of Group I hydrogenases for hydrogen uptake, likely exhibiting different hydrogen affinities (Grote et al., 2012; Labrenz et al., 2013). Group II hydrogenases likely participate in hydrogen sensing or energy conversion at a low concentration of hydrogen (Campbell et al., 2009; Sievert et al., 2008b; Han and Perner, 2015). Previous study showed that Group II hydrogenases were not found in the hydrothermal vent *Sulfurimonas* isolates, but present in the marine water and sediments, thus speculated that in these habitats hydrogen concentration is relatively low than in hydrothermal vents, and that this group of hydrogenases may be more important under low hydrogen concentrations (Han and Perner, 2015). Yet, in this study, Group II hydrogenase was found in vent *Sulfurimonas* species including strains NW10^T^, NW8N, and S2-6. Hence, it is unlikely that Group II hydrogenase in vent *Sulfurimonas* is specialized to function at low hydrogen concentrations, and the elucidation of the role of Group II hydrogenase in *Sulfurimonas* species will require examination of its activity under different hydrogen concentrations.

Additionally, four *Sulfurimonas* species harbored Group IV hydrogenases, possibly involved in hydrogen evolution or energy conversion (Vignais and Billoud, 2007). In addition to Hyc and Ech, Coo subtype of group IV hydrogenase was first found in vent strain *Sulfurimonas* sp. S2-6, containing the cluster *CooLUHF* (Table 3). This energy-converting hydrogenases can couple CO and H_2_ metabolism with energy conservation. The Coo hydrogenase cluster has been identified in 30 bacterial representatives, at particularly high frequency in Deltaproteobacteria (many sulfate reducers, e.g., *Desulfovibrio vulgaris*), Alpha- (e.g., *Rhodospirillum rubrum*) and Campylobacteria (*Nautilia profundicola*), Firmicutes (e.g., *Carboxydothermus hydrogenoformans*), Betaproteobacteria and Gammaproteobacteria (Schoelmerich and Müller, 2019). Phylogenetic analysis based on the large subunit CooH indicated that this hydrogenase was clustered together with those of *R. rubrum*, *D. vulgaris* and *C. hydrogenoformans* (Figure 6), which have been experimentally demonstrated to oxidize CO, producing carbon dioxide and hydrogen as products, through CooMKLXUHF complexes (Bonam and Ludden, 1987; Wu et al., 2005; Caffrey et al., 2007). Further, protein domain analyses by DELTA-BLAST and PSI-BLAST in NCBI showed that CooH contains three functional domains: two respiratory-chain NADH dehydrogenases and one nickel-dependent hydrogenase. The Coo hydrogenases could be involved in H_2_ production from CO according to the overall equation CO + H_2_O→CO_2_ + H_2_ (Heidelberg et al., 2004). In any case, this is the first report of Coo hydrogenase found in the genus *Sulfurimonas* and the function of H_2_ production from Coo hydrogenase in *Sulfurimonas* species needs further experimental confirmation.

In a word, the evolution and functions of different types of Sqr and hydrogenases within one host remain enigmatic and need further investigations, especially to elucidate their relevance in relation to host adaptation to hydrothermal environments. In addition to energy metabolism, comparative genome analysis revealed other vent-specific gene signatures related to signal transduction mechanisms and inorganic ion transporter mechanisms, including unique two-component signal transduction system and a relative abundance of signaling proteins, the ABC transporter system and metal ion transporter (such as Zn^2+^, Mg^2+^, Cu^2+^ and K^+^ transporter), and outer membrane protein TolC. Overall, this versatile energy metabolism, environmental sensing systems, and multiple transporter mechanisms could contribute to the wide spreading and high adaptability of these organisms to different hydrothermal vent fields globally.

## Conclusion

Strain NW10^T^ represents a novel species named as *S. hydrogeniphila*, which is abundant (≥1%) in nearly half of deep-sea hydrothermal vent environments globally. It differs from other established species of this genus in that it can produce a large amount of extracellular sulfur during thiosulfate oxidation. This discovery highlights that the role in hydrothermal vent ecosystems worth further investigations. Strain NW10^T^ can grow with a variety of electron donors (various sulfur compounds and hydrogen) and acceptors (nitrate, oxygen, and elemental sulfur), with a preference for hydrogen utilization. Correspondingly, its genome encodes very diverse energy metabolisms for carbon fixation, with sulfur oxidation coupled nitrate/oxygen reduction, or hydrogen oxidation coupled the reduction of nitrate, oxygen, and even elemental sulfur, showing its adaptability to fluctuating hydrothermal vent environments. Comparative genomics revealed unique genes encoding Type V Sqr, Group II and Coo hydrogenases that may facilitate vent *Sulfurimonas* survival in deep-sea hydrothermal environments.

### Description of *Sulfurimonas hydrogeniphila* sp. nov.

*Sulfurimonas hydrogeniphila* (hy.dro.ge.ni.phi’la. N.L. neut. n. *hydrogenum* hydrogen; Gr. adj. *philos* loving; N.L. fem. adj. *hydrogeniphila* hydrogen-loving, because growth prefers hydrogen).

Cells are Gram-negative short rods (0.8–3.5 × 0.4–0.8 μm) that are motile by means of a polar flagellum. Anaerobic to aerobic, even with the air in the headspace (optimum 6%). Growth occurs at 4-45°C (optimum 33°C), pH 5.0–9.0 (optimum pH 6.0–6.5), and with 2.0–4.0% (w/v) NaCl concentration (optimum 3.0%). Obligate chemolithoautotrophic growth occurs with H_2_, thiosulfate, sulfide, elemental sulfur as electron donors, and oxygen, nitrate, and S^0^ can be utilized as an electron acceptor. Organic substrates are not utilized as carbon sources and energy sources. Major cellular fatty acids are C_16__:__1_ ω7c (31.6%), C_16__:__0_ (28.2%) and C_18__:__1_ ω7c (18.4%).

The type strain, NW10^T^ (=MCCC 1A13987^T^ = KTCC 15781^T^) was isolated from the deep-sea hydrothermal sulfide chimneys in the Carlsberg Ridge of Northwest Indian Ocean. The G + C content of its genomic DNA is 37.3 mol%.

## Materials and Methods

### Sample Collection and Enrichment

The chimney samples were collected near an active hydrothermal vent on the Carlsberg Ridge (60°31′E, 6°21′N), at a depth of 2936 m, by a human operated vehicle “Jiao-long” during COMRA DY 38 oceanic scientific cruise in March 2017. Aboard the research vessel *Xiang-Yang-Hong No. 9*, chimney samples were immediately transferred into MMJHS medium under a gas phase mixture of 80% H_2_/18% CO_2_/2% O_2_ (200 kPa) and then incubated at 28°C according to the previous description (Inagaki et al., 2003). After successful enrichment with MMJHS medium, the well-grown culture was further purified using the dilution-to-extinction technique with the same medium. MMJS medium consisted of NaCl (30 g l^–1^), KCl (0.33 g l^–1^), NH_4_Cl (0.25 g l^–1^), MgCl_2_⋅6H_2_O (4.18 g l^–1^), CaCl_2_⋅2H_2_O (0.14 g l^–1^), K_2_HPO_4_ (0.14 g l^–1^), NaHCO_3_ (1 g l^–1^), Na_2_S_2_O_3_⋅5H_2_O (10 mM), Wolfe’s vitamins (1 ml l^–1^) and trace element solution (10 ml l^–1^).

### Phylogenomic Analysis

The genomic DNA was prepared according to the method described previously (Jiang et al., 2010) and the 16S rRNA gene was amplified by PCR primers described previously (Lane, 1991). The sequence was compared with those of other type strains using the EzTaxon-e server (Yoon et al., 2017). The 16S rRNA gene sequences of the related taxa were obtained from the GenBank database. Phylogenetic trees were constructed by using MEGA 6.0 (Tamura et al., 2013) using the neighbor-joining (Saitou and Nei, 1987), maximum-likelihood (Felsenstein, 1981) and minimum evolution methods (Rzhetsky and Nei, 1992) after multiple alignments of the data by CLUSTAL_W. Evolutionary distances were calculated using Kimura’s two-parameter model and bootstrap values were determined based on 1,000 replications. The phylogenomic tree was constructed based on an up-to-date 92 bacterial core gene sets by UBCG version 3.0 (Na et al., 2018). Genome sequences of reference taxa were retrieved from the NCBI database and the 92 concatenated core genes were extracted, aligned and concatenated using default parameters. The tree topology was supported by the maximum-likelihood method for 100 bootstrap replications using RAxML version 8.2.11 (Stamatakis, 2014) with the GTR + CAT model.

### Phenotypic and Chemotaxonomic Characterization

Cell morphology was observed under a transmission electron microscopy (Model JEM-1230, JEOL, Japan) with cultures grown in MMJHS medium at 28°C for 1 day. The physiological characterization of the isolate was tested on MMJHS medium (Inagaki et al., 2003). After autoclaving, the medium (10 ml) was dispensed into 50 ml serum bottles, then sealed with a butyl-rubber stopper under a gas phase of 80% H_2_/18% CO_2_/2% O_2_ (200 kPa). All cultivation experiments were performed in triplicate, unless otherwise specified. The growth was measured by direct cell counting using a phase contrast microscope (Eclipse 80i, Nikon, Japan). Growth at different temperatures was examined in MMJHS at 4, 10, 15, 20, 25, 28, 30, 33, 35, 37, 45, 50, and 60°C. The growth salinity range was examined by adjusting the concentrations of NaCl between 0 and 9.0% (w/v), at 0.5 (w/v) intervals. To determine the effect of pH on growth, the pH of MMJHS medium was adjusted from 4.5 to 9.0 with a 0.5 pH unit interval by using 30 mM acetate/acetic acid buffer (pH 4.0–5.0), MES (pH 5.0–6.0), PIPES (pH 6.0–7.0), HEPES (pH 7.0–7.5), Tris and CAPSO (pH 8.0 and above). The effect of O_2_ on growth was examined by adjusting the oxygen concentration (0, 1, 2, 4, 6, 8, 10% at 200 kPa and 20% at 100 kPa) in the headspace gas. To test the anaerobic growth, 10 mM nitrate was added as an alternative electron acceptor.

The ability of sulfur oxidation of strain NW10^T^ was tested using the following sulfur compounds as the sole energy source in the MMJS medium, including thiosulfate (10 mM), sulfite (5 mM), thiocyanate (5 mM), tetrathionate (5 mM), elemental sulfur (1% w/v), or sodium sulfide (50, 100, 500 μM, and 1 mM) with a gas phase of 74% N_2_/20% CO_2_/6% O_2_ (200 kPa). Molecular hydrogen was also tested in MMJH medium in the absence of thiosulfate under a gas phase of 74% H_2_/20% CO_2_/6% O_2_ (200 kPa). To determine the utilization of other electron acceptors, each of the potential electron acceptors, such as thiosulfate (10 mM), tetrathionate (10 mM), sulfite (2 mM and 10 mM), elemental sulfur (1%, w/v), nitrate (10 mM), nitrite (1 mM and 5 mM), selenate (5 mM), arsenate (5 mM), fumarate (10 mM), and ferric citrate (20 mM) was examined with MMJHS medium under 80% H_2_/20% CO_2_ (200 kPa). Heterotrophic growth was examined in MMJHS medium in the absence of NaHCO_3_ under a gas phase of 94% N_2_/6% O_2_ (200 kPa). Each of the following potential organic carbon compounds was tested: 0.1% (w/v) peptone, starch, tryptone, yeast extract, casein, and casamino acids, 5 mM formate, acetate, propionate, citrate, tartrate, fumarate, succinate, malate, and pyruvate, 5 mM of each of 20 amino acids, 0.02% (w/v) glucose, galactose, sucrose, fructose, lactose, maltose, and trehalose. In an attempt to determine the alternative energy source, these organic compounds were used as an energy source in MMJ medium to replace thiosulfate under a gas phase of 74% N_2_/20% CO_2_/6% O_2_ (200 kPa).

For analyses of fatty acids, cells grown on MMJHS medium at 33°C for 24 h were saponified, methylated, and extracted following the standard MIDI protocol (Sherlock Microbial Identification System, version 6.0B). The fatty acids were analyzed by gas chromatography (Agilent Technologies 6850) and then the result was identified using the TSBA6.0 database of the Microbial Identification System.

### Scanning Electron Microscopy and Raman Spectromicroscopy of Extracellular Sulfur

Scanning electron microscope (SEM) (S-3400N; Hitachi, Japan) and Raman spectra (XploRA; Horiba JY, France) were used to identify the shape, components and structure of extracellular sulfur produced by strain NW10^T^. For SEM analysis, a milky white suspension was collected using polycarbonate filters (Merck Millipore, pore size 3.0 μm), rinsed three times with deionized water and observed by SEM at 5 kV. Energy-Dispersive Spectrum (EDS) (model 550i, IXRF systems, United States) equipment with SEM was employed at an accelerating voltage of 5 keV for 30 s. For Raman analysis, about 5 ml samples were concentrated by centrifugation and rinsed three times with deionized water. The supernatant was decanted and the pellet was lyophilized overnight. A small amount of powder was put on the glass slide and observed by microconfocal method. Raman spectra were collected using a Horiba XploRA Raman spectrometer coupled with a 532 nm laser source. For α-S_8_ standard, commercial precipitated sulfur (purity > 99%, Thermo Fisher Scientific) was used.

### Whole Genome Sequence Analysis

The complete genome of strain NW10^T^ was sequenced by Tianjin Biochip Corporation (Tianjin, PR China), using the single molecule real-time (SMRT) technology on the Pacific Biosciences (PacBio) sequencing platform. The sequenced reads were filtered, and high quality paired-end reads were assembled to construct a circular genome with SOAPdenovo (version 2.04)^1^. The G + C content of the chromosomal DNA was determined according to the genome sequence. Gene prediction was carried out by Glimmer program (Delcher et al., 2007). rRNA identification was performed with the RNAmmer 1.2 software (Lagesen et al., 2007), and tRNAscan-SE (version 1.21) was used to identify the tRNA genes (Schattner et al., 2005). Gene prediction and annotation were carried out using NCBI Prokaryotic Genomes Annotation Pipeline (PGAP) and the Rapid Annotation using Subsystem Technology (RAST) pipeline^2^ (Aziz et al., 2008). The functional annotation and metabolic pathways were analyzed by searching against KEGG and COG databases. To further clarify the genetic relatedness between strain NW10^T^ and related species of the genus *Sulfurimonas*, the average nucleotide identity (ANI) value between two genomes was calculated using the web service of EZGenome^3^ (Richter and Rosselló-Móra, 2009). The predicted *in silico* DNA-DNA hybridization (DDH) values were determined online^4^ using the Genome-to-Genome Distance Calculator (GGDC) (Auch et al., 2010).

### Comparative Genomics Analyses

To avoid the possible deviations due to different annotation methods, we used RAST server for re-annotation. A pan-genome for the eleven genomes was determined by BPGA (Chaudhari et al., 2016) pipeline to identify orthologous groups among *Sulfurimonas* strains and to extrapolate the pan-genome models of applying default parameters. Orthologous clusters were assigned by grouping all protein sequences in the 11 genomes using USEARCH based on their sequence similarity (*E*-value < 10^–5^, >50% coverage) and each protein was assigned to one protein family. The pan genome analysis complied the set of core genes shared among all strains, a set of distributed genes shared with more than two but not all strains, and unique genes only found in a single strain. COG and KEGG distributions of the core, distributed and unique gene families were calculated based on representative sequences.

## Data Availability Statement

The datasets presented in this study can be found in online repositories. The names of the repository/repositories and accession number(s) can be found in the article/Supplementary Material.

## Author Contributions

SW, LJ, SY, and ZS designed the study. ZS contributed to deep sea sampling and conducted on board experiments. SW and QH conducted the experiments and analyzed the data. SW, LJ, and LC drafted the manuscript. BZ, XF, QL, and ZS revised the manuscript. All authors read and approved the final manuscript.

## Conflict of Interest

The authors declare that the research was conducted in the absence of any commercial or financial relationships that could be construed as a potential conflict of interest.

## Footnotes

1 http://soap.genomics.org.cn/

2 http://rast.nmpdr.org/

3 www.ezbiocloud.net/tools

4 http://ggdc.dsmzde/distcalc2.php

## Supplementary Material

Supplementary Figure 1 Transmission electron micrograph of cells of *S. hydrogeniphila* NW10^T^. Bar, 2.0 μm.

Supplementary Figure 2 Neighbor-joining phylogenetic tree based on 16S rRNA gene sequences showing the relationship of strain NW10^T^ with other members within the genus *Sulfurimonas*. Bootstrap values based on 1,000 replicates are shown at branch nodes. Bar, 0.01 substitutions per nucleotide position.

Supplementary Figure 3 Minimum evolution phylogenetic tree based on 16S rRNA gene sequences showing the relationship of strain NW10^T^ with other members within the genus *Sulfurimonas*. Bootstrap values based on 1,000 replicates are shown at branch nodes. Bar, 0.01 substitutions per nucleotide position.

Supplementary Figure 4 Circular diagrams of the *S. hydrogeniphila* NW10 chromosomes displaying relevant genome features. The following information is provided from outside to inside: The first circle and the fourth circle are protein coding sequences on the forward and reverse strand; The second and third circles are CDS, tRNA and rRNA on the forward and reverse stand, respectively. The fifth circle is the GC content (swell outward/inward indicates higher/lower G + C compared with the average G + C content); The sixth circle is the GC-Skew value (purple/yellow indicate positive/negative values) and the innermost circle identifies the genome size.

Supplementary Table 1 The specific information of key enzymes involved in sulfur, nitrogen, hydrogen, and carbon metabolisms in 11 *Sulfurimonas* species based on RAST annotations.

Supplementary Table 2 Vent-specific genes and their functional annotations. Annotation is the functional annotation based on the SEED database. COG_sutype, shows the functional classification based on COG family assignments.

## Abbreviations

MCCC: Marine Culture Collection of China
KCTC: Korean Collection for Type Cultures
ANI: average nucleotide identity
DDH: DNA-DNA hybridization.
