# Supplementary figures and images for: Characterization of Sulfurimonas hydrogeniphila sp. nov., a Novel Bacterium Predominant in Deep-Sea Hydrothermal Vents and Comparative Genomic Analyses of the Genus Sulfurimonas
Source: Front Microbiol. 2021 Feb 26;12:626705. doi: 10.3389/fmicb.2021.626705 (PMC7952632; doi:10.3389/fmicb.2021.626705)

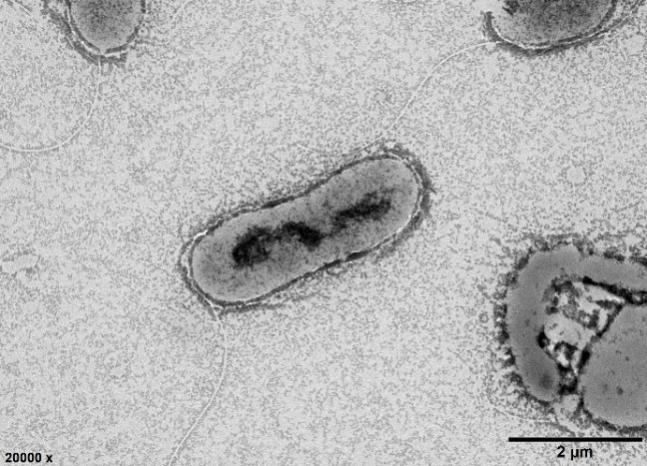

Supplement: Supplementary Figure 1 — Transmission electron micrograph of cells of S. hydrogeniphila NW10T. Bar, 2.0 μm. [file Image_1.TIF]

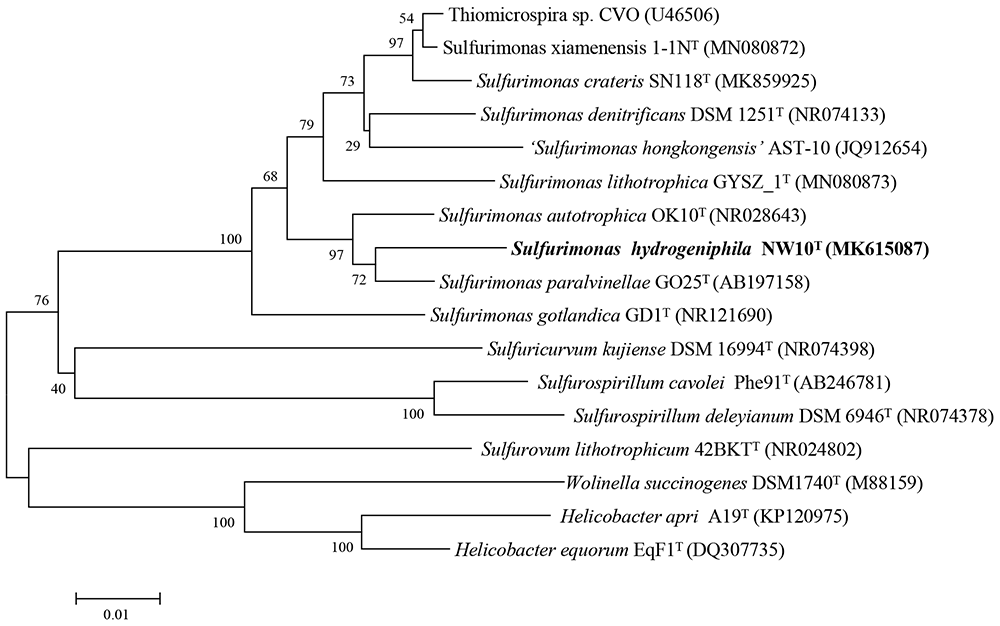

Supplement: Supplementary Figure 2 — Neighbor-joining phylogenetic tree based on 16S rRNA gene sequences showing the relationship of strain NW10T with other members within the genus Sulfurimonas. Bootstrap values based on 1,000 replicates are shown at branch nodes. Bar, 0.01 substitutions per nucleotide position. [file Image_2.TIF]

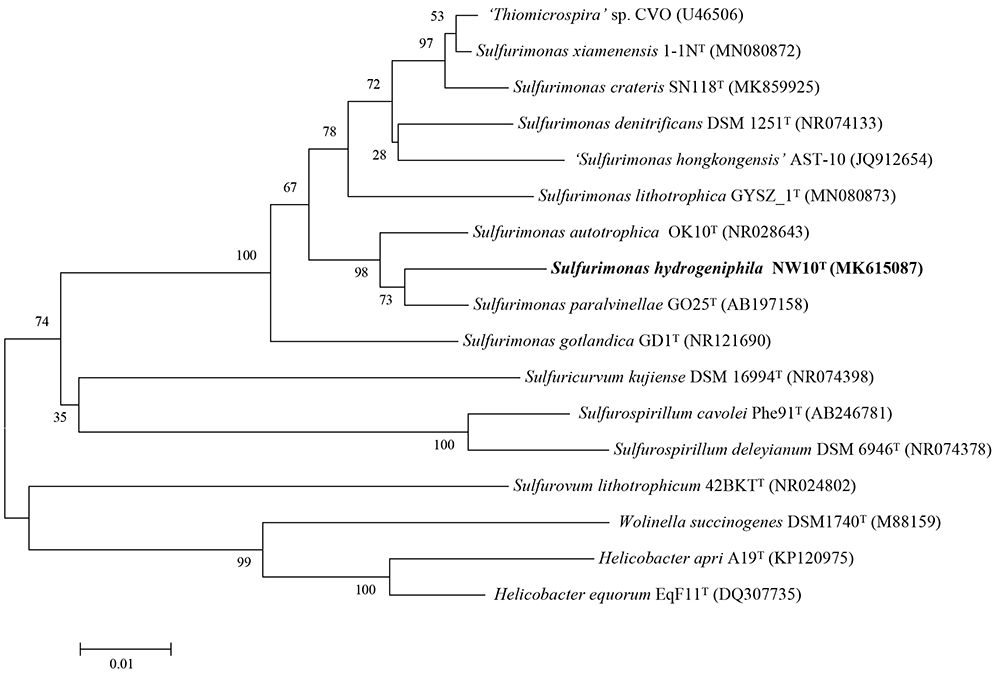

Supplement: Supplementary Figure 3 — Minimum evolution phylogenetic tree based on 16S rRNA gene sequences showing the relationship of strain NW10T with other members within the genus Sulfurimonas. Bootstrap values based on 1,000 replicates are shown at branch nodes. Bar, 0.01 substitutions per nucleotide position. [file Image_3.TIF]

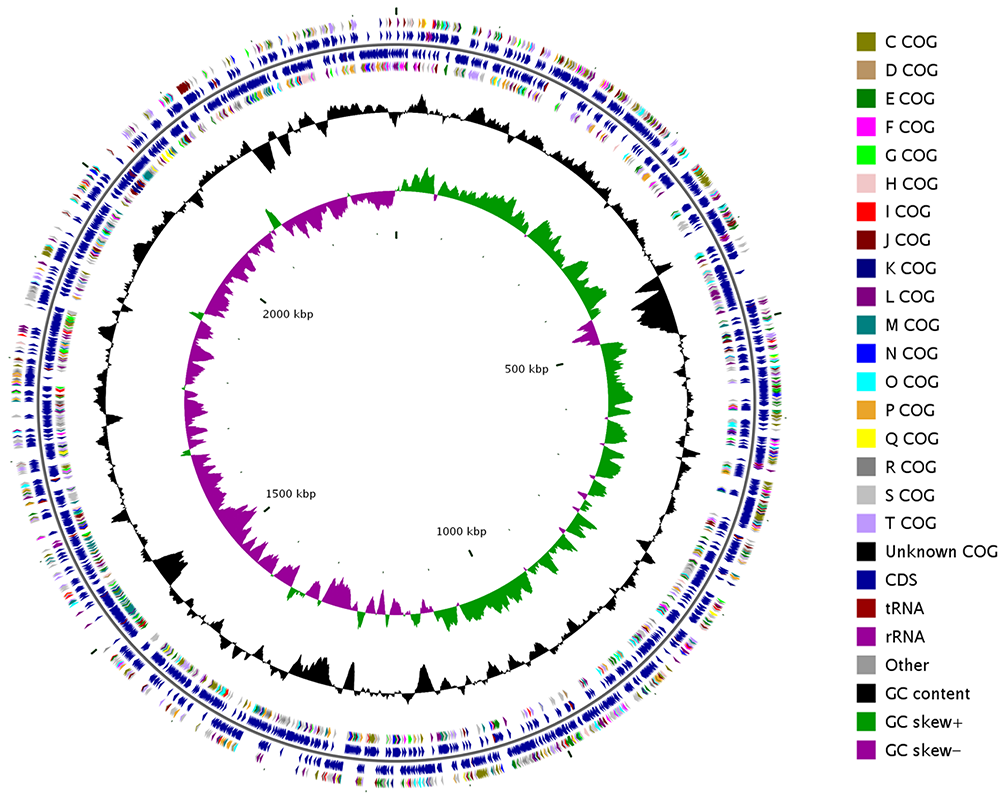

Supplement: Supplementary Figure 4 — Circular diagrams of the S. hydrogeniphila NW10 chromosomes displaying relevant genome features. The following information is provided from outside to inside: The first circle and the fourth circle are protein coding sequences on the forward and reverse strand; The second and third circles are CDS, tRNA and rRNA on the forward and reverse stand, respectively. The fifth circle is the GC content (swell outward/inward indicates higher/lower G + C compared with the average G + C content); The sixth circle is the GC-Skew value (purple/yellow indicate positive/negative values) and the innermost circle identifies the genome size. [file Image_4.TIF]
